# Global ecological associations between overweight/obesity and non-communicable disease prevalence among children aged 5–9 years: a sex-stratified analysis of 174 countries

**DOI:** 10.3389/fpubh.2026.1921938

**Published:** 2026-08-26

**Authors:** Wenyu Ye, Li Shi, Sha Ye, Yuanqian Wen, Jiayi Gong, Wei Qian, Li Zhang, Lili Lu

**Affiliations:** 1 Department of Pediatrics, Longhua Hospital, Shanghai University of Traditional Chinese Medicine, Shanghai, China; 2 Department of Preventive Medicine, School of Public Health, Shanghai University of Traditional Chinese Medicine, Shanghai, China; 3 Institute of Digestive Diseases, Longhua Hospital, Shanghai University of Traditional Chinese Medicine, Shanghai, China

**Keywords:** childhood obesity, children, ecological study, epidemiology, global health, non-communicable diseases, overweight

## Abstract

**Introduction:**

Childhood overweight and obesity are increasing worldwide, yet their population-level associations with a broad range of non-communicable diseases (NCDs) among children aged 5–9 years remain insufficiently characterized. This study examined the country-level associations between overweight/obesity and NCDs prevalence and explored whether these association patterns differed by sex.

**Methods:**

This ecological study included 2022 national-level data from 174 countries. The prevalence of NCDs among children aged 5–9 years was obtained from the Global Burden of Disease (GBD), while overweight and obesity prevalence estimates were obtained from the World Health Organization Global Health Observatory (GHO). We utilized Spearman’s correlation analysis to assess correlations and Random Forest to explore predictive importance, while generating forest plots and importance summary plots as independent visualization tools for the results.

**Results:**

Significant global disparities in childhood overweight/obesity existed, with high prevalence in the Americas/Europe and low in Africa/Southeast Asia. Both were significantly positively correlated with multiple NCDs (*p* < 0.05). For overweight and obesity, the strongest correlations were with neoplastic disease, digestive and urinary system disease, neurological and psychiatric disease, as well as cardiovascular disease. After Benjamini-Hochberg false discovery rate (FDR) correction, multiple positive associations remained significant in the overall and boys’ analyses, including associations with skin-related diseases among boys, whereas no positive associations remained significant among girls.

**Conclusion:**

This study provides epidemiological evidence on robust positive associations between childhood overweight/obesity and a broad spectrum of NCDs. Governments and global health systems should implement preventive measures against childhood weight abnormalities.

## Graphical abstract

**Figure:**
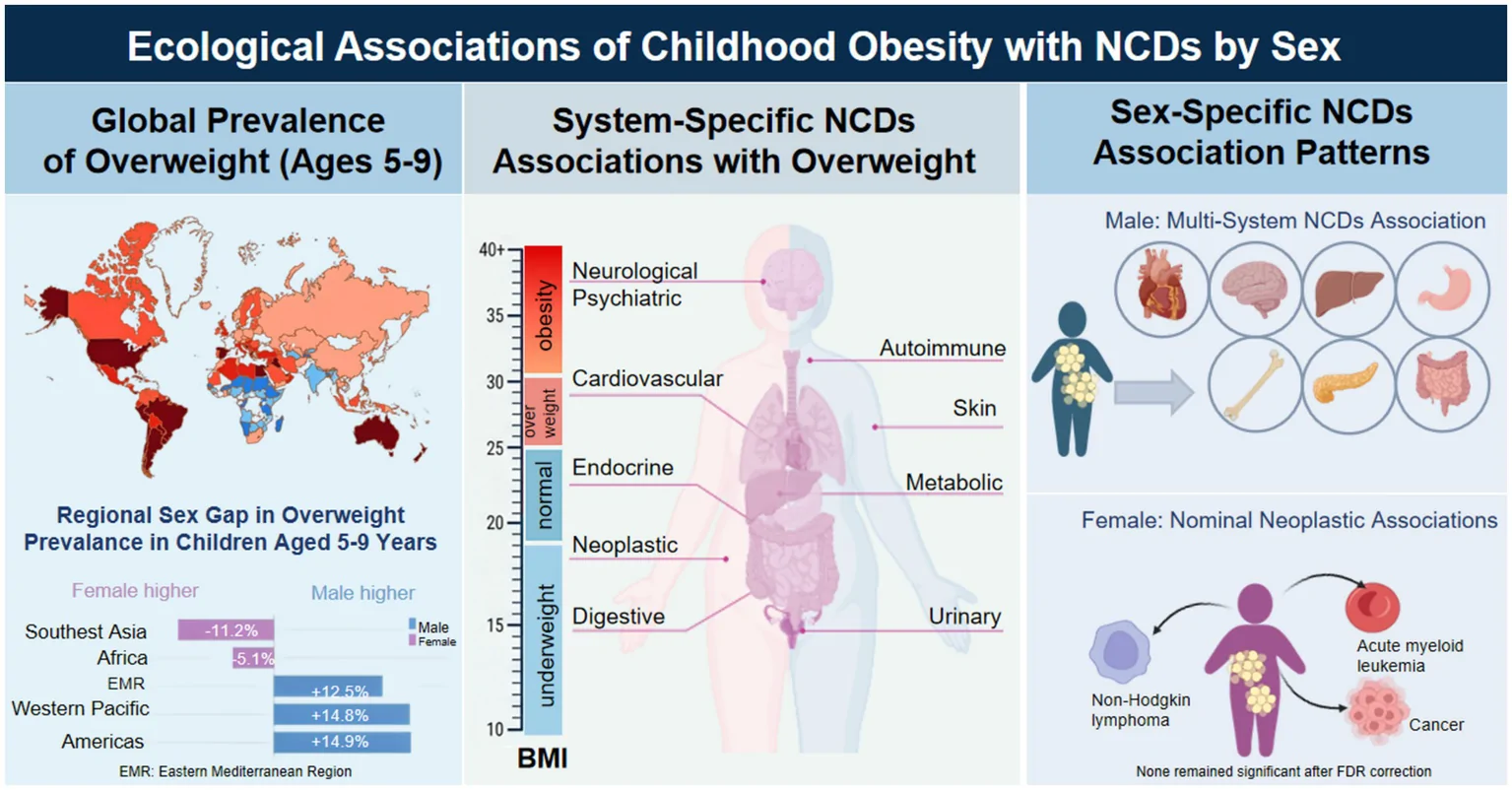

## 1 Introduction

The global prevalence of overweight and obesity among children aged 5–9 years has increased substantially over the past three decades, making childhood overweight/obesity a major global public health concern (1–4). Since the early 1990s, when the global prevalence of overweight and obesity in this age group was below 10% and 3%, respectively, both conditions have increased substantially. By 2022, these rates reached approximately 25% for overweight and 8.5% for obesity. Excess weight during childhood is associated with adverse physical and psychological health outcomes and may persist into adolescence and adulthood, thereby contributing to long-term health burdens (5–8). These trends underscore the importance of understanding the health conditions that coexist with overweight and obesity during early childhood and identifying opportunities for timely surveillance and prevention (9).

Epidemiological studies consistently indicate that a high body mass index (BMI) is a modifiable risk factor for a growing number of health conditions (10, 11). These include early-onset cardiovascular abnormalities, insulin resistance (a precursor to type 2 diabetes), increased risk of chronic kidney disease, heightened susceptibility to certain cancers later in life, and musculoskeletal disorders such as osteoarthritis (12, 13). Collectively, these conditions significantly contribute to childhood morbidity and long-term adverse health outcomes (6, 7). Several biological pathways may underlie the associations between excess adiposity and multisystem health conditions. Adipose tissue dysfunction may be accompanied by chronic low-grade inflammation, insulin resistance, altered adipokine secretion, oxidative stress, endothelial dysfunction, and unfavorable hemodynamic changes, which may be relevant to cardiovascular, endocrine, renal, gastrointestinal, neurological, and other chronic conditions (7, 12, 14). However, these mechanisms have mainly been proposed in previous individual-level and experimental studies and were not directly examined in the present ecological analysis. Consequently, precise and timely data on the prevalence of weight abnormalities and their multi-systemic impact are essential to guide evidence-based prevention policies. Previous studies have focused predominantly on metabolic conditions such as hypertension and type 2 diabetes, and comprehensive evidence regarding the associations between childhood overweight/obesity and a broad spectrum of NCDs among children aged 5–9 years remains limited (15). In particular, few studies have examined these associations across a large number of countries using comparable national-level data. Moreover, potential differences between boys and girls have not been adequately characterized at the global level. Sex-related differences in body composition, fat distribution, developmental characteristics, and inflammatory responses may contribute to different patterns of association between weight status and health outcomes (16–20). Therefore, sex-stratified analyses may provide additional information beyond analyses of the overall population.

This study integrates data from the 2022 GHO on the prevalence of overweight and obesity among children aged 5–9 years both overall and by sex, with 2022 GBD data on the prevalence of major NCDs in the same age group and by sex. Through a cross-national analysis, the study explores correlations between the prevalence of childhood overweight or obesity and the prevalence of specific NCDs. It further examines whether these patterns of association differ for both sexes. This research reflects current global child health challenges, and may provide a reference for sex-disaggregated child health surveillance, population-level prevention planning, and the prioritization of future longitudinal and individual-level research.

## 2 Methods

### 2.1 Data source

Data on the prevalence of NCDs in the total population aged 5–9 years were extracted from the GBD database (21). For the prevalence of obesity and overweight in the same age group, we retrieved data from the GHO Database. A total of 174 countries with complete 2022 data in both databases were included in the final analysis, while countries with missing data in either database were excluded.

### 2.2 Outcomes and variables

The outcome variables were the prevalence of multiple NCDs among children aged 5–9 years, stratified by sex. These NCDs cover diseases of multiple systems: neoplastic, cardiovascular, digestive and urinary system, neurological and psychiatric, genetic, skin, endocrine and metabolic, and autoimmune. The exposure variables were the prevalence of overweight and obesity among children aged 5–9 years. Overweight and obesity were defined according to the WHO BMI-for-age reference for children aged 5–19 years. Overweight was defined as BMI-for-age greater than +1 standard deviation above the WHO reference median, and obesity was defined as BMI-for-age greater than +2 standard deviations. All variables were national-level prevalence data, and only countries with complete data in both databases were included for final analysis.

### 2.3 Statistical analysis

As the data were not normally distributed, a non-parametric Spearman’s rank-order correlation analysis was performed to assess the correlation between the prevalence of NCDs and overweight/obesity. These correlation analyses were presented by the correlation coefficient (*r*) and 95% confidence interval (CI), using IBM SPSS 27.0 (IBM Corp.) with a two-sided *p* < 0.05 indicating statistical significance. To account for multiple comparisons, the FDR correction was applied, and an FDR-adjusted *q* value < 0.05 was considered statistically significant after correction.

Random Forest regression was used as a complementary exploratory analysis to identify potential nonlinear predictive patterns that might not be captured by Spearman correlation (22). A separate model was fitted for each NCD outcome, with national overweight or obesity prevalence as the sole predictor and the corresponding NCD prevalence as the continuous outcome. The analyses were restricted to NCD outcomes that remained statistically significant after FDR correction. Models were fitted in R version 4.5.1 using the random Forest package with 500 trees and mtry = 1 (23); other parameters were retained at their default settings. Complete-case analysis was performed for each model, and internal validation was based on out-of-bag observations. Model performance was evaluated using OOB RMSE, MAE, and *R*^2^. Predictor importance was measured using permutation-based %IncMSE. Importance values were interpreted as model-specific exploratory predictive measures rather than effect estimates, individual-level risks, or evidence of causality.

The analyses were unadjusted because harmonized country-level covariate data were not incorporated into the present dataset. Therefore, the observed associations may be influenced by residual and unmeasured confounding. The study design, database matching, outcome selection, and overall analytical workflow are summarized in Supplementary Figure S1.

## 3 Results

### 3.1 Global and regional prevalence of overweight and obesity among children aged 5–9 years

The global distribution and regional variation of overweight and obesity prevalence in 5–9 years old children are illustrated in Supplementary Figure S2. Geographically, Chile had the highest prevalence of both overweight and obesity, at 63.47% and 35.75%, respectively (Supplementary Figures S2A,B). At the continental level, the overweight burden is most pronounced in Americas, Europe, and Western Pacific, while Africa and Southeast Asia exhibit lower prevalence (Supplementary Figure S2C).

**Figure 1:**
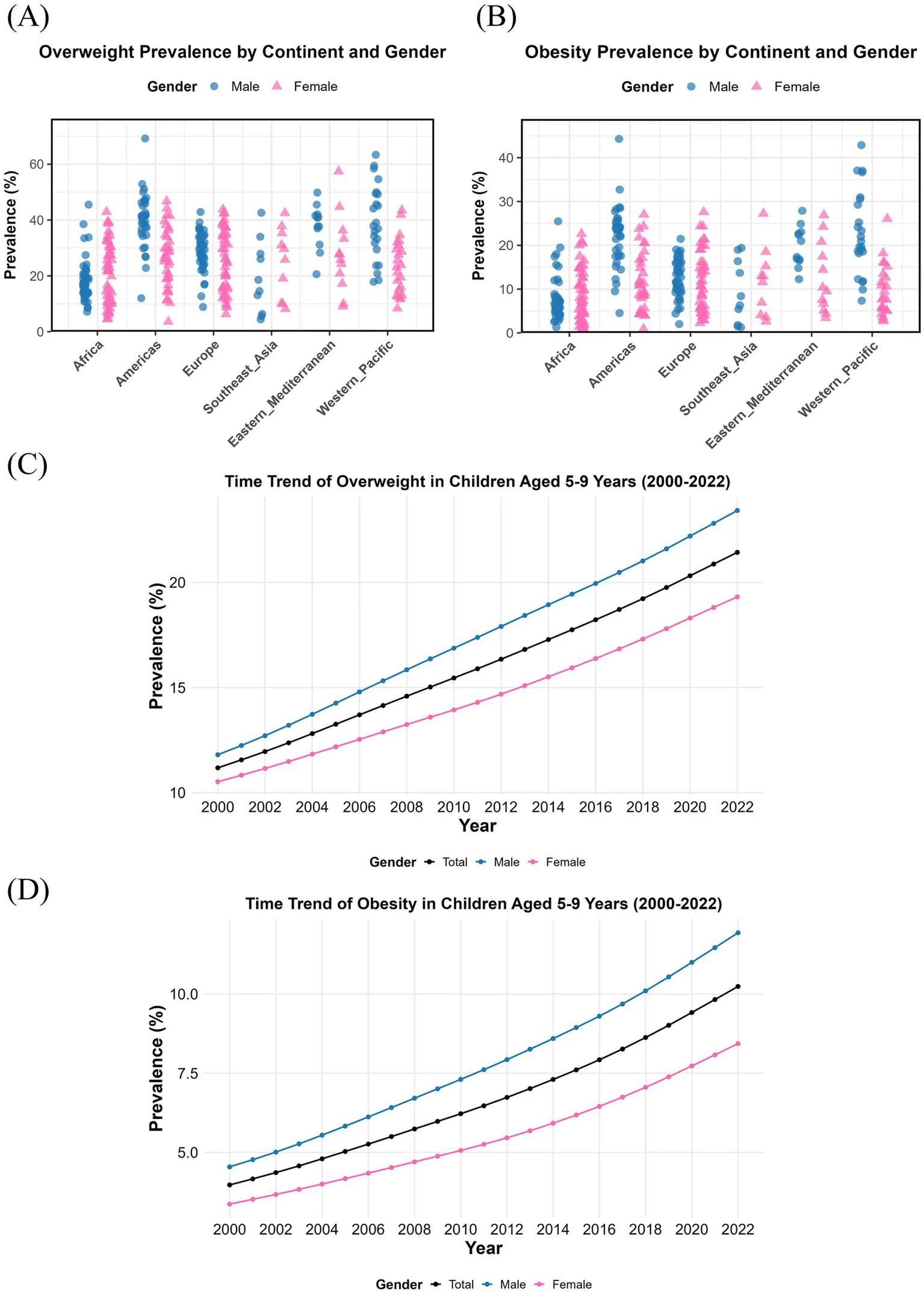
Gender-specific prevalence of overweight and obesity by continent and global temporal trends. **(A)** Overweight prevalence in boys and girls across continents. **(B)** Obesity prevalence in boys and girls across continents. **(C)** Global trends in overweight prevalence from 2000 to 2022. **(D)** Global trends in obesity prevalence from 2000 to 2022.

Stratified by sex, a distinct geographic pattern emerged: the higher prevalence of overweight among males was predominantly observed in Americas and Western Pacific, whereas a higher prevalence of overweight among females was primarily evident in Africa. The regional distribution of obesity exhibited sex-specific disparities consistent with those documented for overweight (Figures 1A,B). From 2000 to 2022, the global prevalence of overweight increased from 11.18% to 21.43%. Overweight prevalence increased from 11.80% to 23.42% among boys and from 10.52% to 19.31% among girls (Figure 1C). Obesity prevalence also increased over this period, from 4.54% to 11.93% among boys and from 3.37% to 8.44% among girls (Figure 1D).

**Figure 2:**
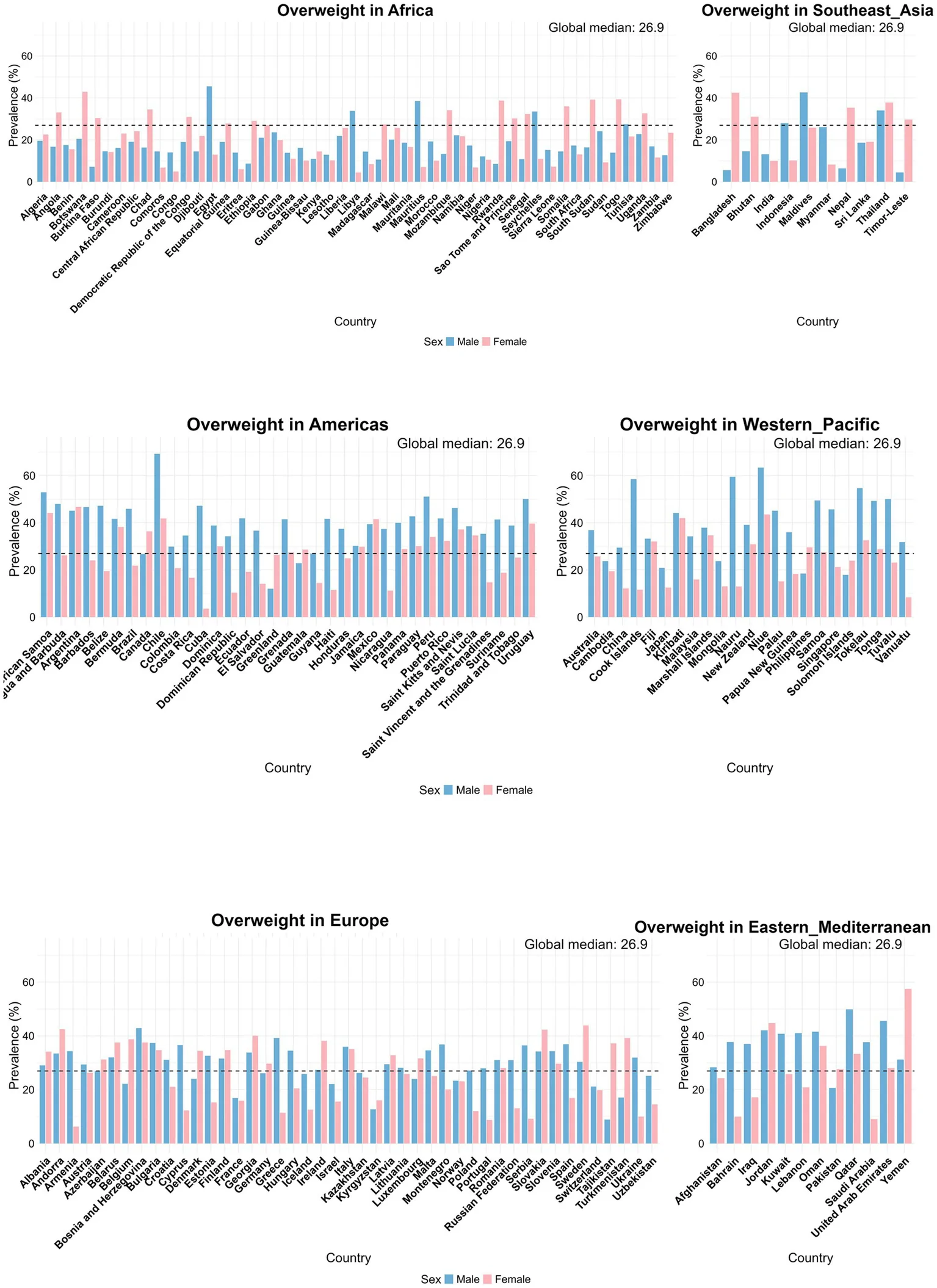
Gender differences in overweight prevalence across continents.

Country- and region-specific overweight prevalence is presented in Figure 2. The highest regional median values were observed in the Eastern Mediterranean, the Americas, and the Western Pacific, whereas lower values were observed in Southeast Asia and Africa. A similar regional pattern was observed for obesity (Supplementary Figure S3), with higher median prevalence among boys than among girls in the Americas and Western Pacific.

### 3.2 Association between overweight and NCDs in children aged 5–9 years

Correlation analyses identified 37 nominally significant positive associations between overweight prevalence and NCD prevalence in the overall population. After FDR correction, 34 associations remained statistically significant (*q* < 0.05). Among them, the analysis mainly focused on these categories of diseases: neoplastic, digestive and urinary system, neurological and psychiatric as well as cardiovascular diseases. For overweight, the strongest correlation is observed with subarachnoid hemorrhage (*r* = 0.740, 95% CI: 0.631–0.817), followed by myelodysplastic, myeloproliferative, and other hematopoietic neoplasms (MDS/MPN; *r* = 0.696, 95% CI: 0.579–0.791) and bulimia nervosa (*r* = 0.683, 95% CI: 0.554–0.768) (Supplementary Figure S4A). To further evaluate each variable associated with overweight, we applied a machine learning approach, specifically the Random Forest to compute feature importance scores for all included variables. According to the Random Forest output, subarachnoid hemorrhage still achieved the highest importance score (importance score: 48.52), followed by motor neuron disease (importance score: 43.63). When the feature importance was aggregated by disease system, neoplastic-related disease exhibited the most substantial contributors across all systems. Subsequent ranking included diseases of digestive and urinary system, neurological and psychiatric disorders, as well as cardiovascular diseases, respectively (Supplementary Figure S4B).

**Figure 3:**
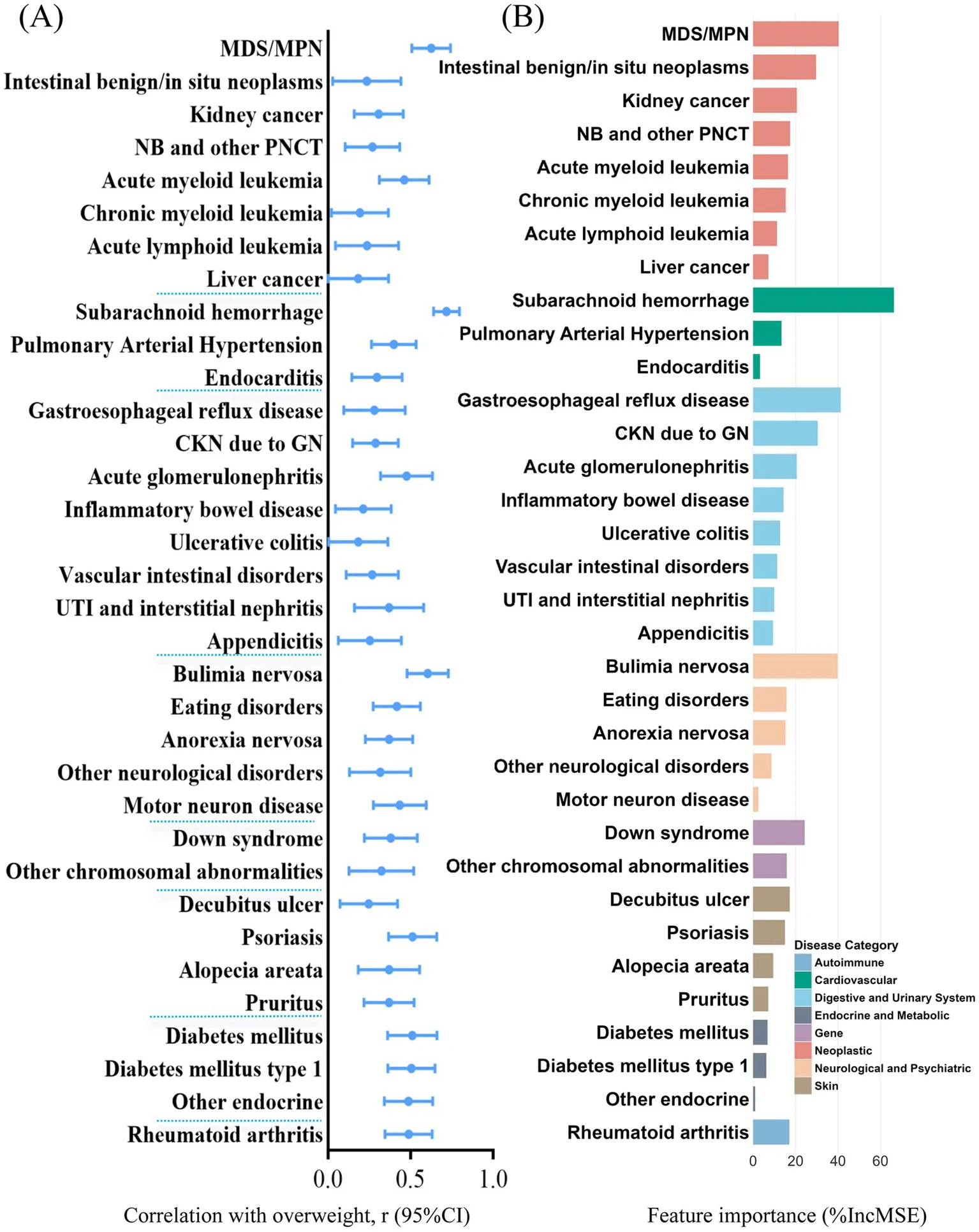
Overweight-NCDs associations and Random Forest feature importance: Analyses stratified by gender. **(A)** Gender-stratified correlation analysis between overweight and NCDs. Only positive associations that remained statistically significant after FDR correction (*q* < 0.05) are shown. **(B)** Gender-stratified Random Forest feature importance for the overweight-NCDs associations. No positive associations remained statistically significant among girls after FDR correction. MDS/MPN: Myelodysplastic, myeloproliferative, and other hematopoietic neoplasms; CNS cancer: Brain and central nervous system cancer; Intestinal benign/*in situ* neoplasms: Benign and in situ intestinal neoplasms; UTI and interstitial nephritis: Urinary tract infections and interstitial nephritis; NB and other PNCT: Neuroblastoma and other peripheral nervous cell tumors; Cervical/uterine benign/in situ neoplasms: Benign and in situ cervical and uterine neoplasms; CKN due to GN: Chronic kidney disease due to glomerulonephritis; Diabetic nephropathy (T1DM): Chronic kidney disease due to diabetes mellitus type 1.

To explore potential sex differences, we next performed a gender-stratified correlation analysis between overweight and NCDs (Figure 3). Among boys, 40 positive associations reached nominal significance, of which 34 remained statistically significant after FDR correction. In detail, the strongest correlations were again observed for subarachnoid hemorrhage (*r* = 0.723, 95% CI: 0.636–0.793), MDS/MPN (*r* = 0.633, 95% CI: 0.503–0.737). Furthermore, three additional diseases were significantly and positively correlated with overweight exclusively in males: psoriasis (*r* = 0.529, 95% CI: 0.358–0.648), other endocrine disorders (*r* = 0.497, 95% CI: 0.336–0.628), and decubitus ulcer (*r* = 0.254, 95% CI: 0.069–0.417). Among girls, three positive correlations reached nominal significance, but none remained statistically significant after FDR correction. Therefore, no Random Forest results are presented for girls (Figure 3A). Further analysis of the sex-stratified patterns showed that, among boys, neoplastic diseases and digestive and urinary system diseases each accounted for 23.5% of all positive associations with overweight, tying for the largest proportion. At the feature level in boys, subarachnoid hemorrhage maintained the highest importance scores (66.33), with gastroesophageal reflux disease (41.26) and bulimia nervosa (39.8) ranking second and third (Figure 3B).

### 3.3 Association between obesity and NCDs in children aged 5–9 years

For obesity, 30 positive associations reached nominal significance in the overall population, of which 26 remained statistically significant after FDR correction (Supplementary Figure S5). Similarly, the analysis mainly focused on following categories of diseases including digestive and urinary system, neoplastic, neurological and psychiatric as well as cardiovascular diseases. Among these, subarachnoid hemorrhage again showing the strongest correlation (*r* = 0.781, 95% CI: 0.698–0.843), a coefficient value in obesity is higher than that observed for overweight. Besides, MDS/MPN (*r* = 0.693, 95% CI: 0.586–0.776) and bulimia nervosa (*r* = 0.615, 95% CI: 0.490–0.716) remain among the top most strongly correlated conditions (Supplementary Figure S5A). In the related feature importance ranking derived from the model, subarachnoid hemorrhage presented the highest importance score (importance score: 56.44), followed by urinary tract infections and interstitial nephritis (UTI and interstitial nephritis; importance score: 43.01), MDS/MPN (importance score: 39.04) were the most prominent, with cardiovascular disease and digestive and urinary system contributing prominently (Supplementary Figure S5B).

**Figure 4:**
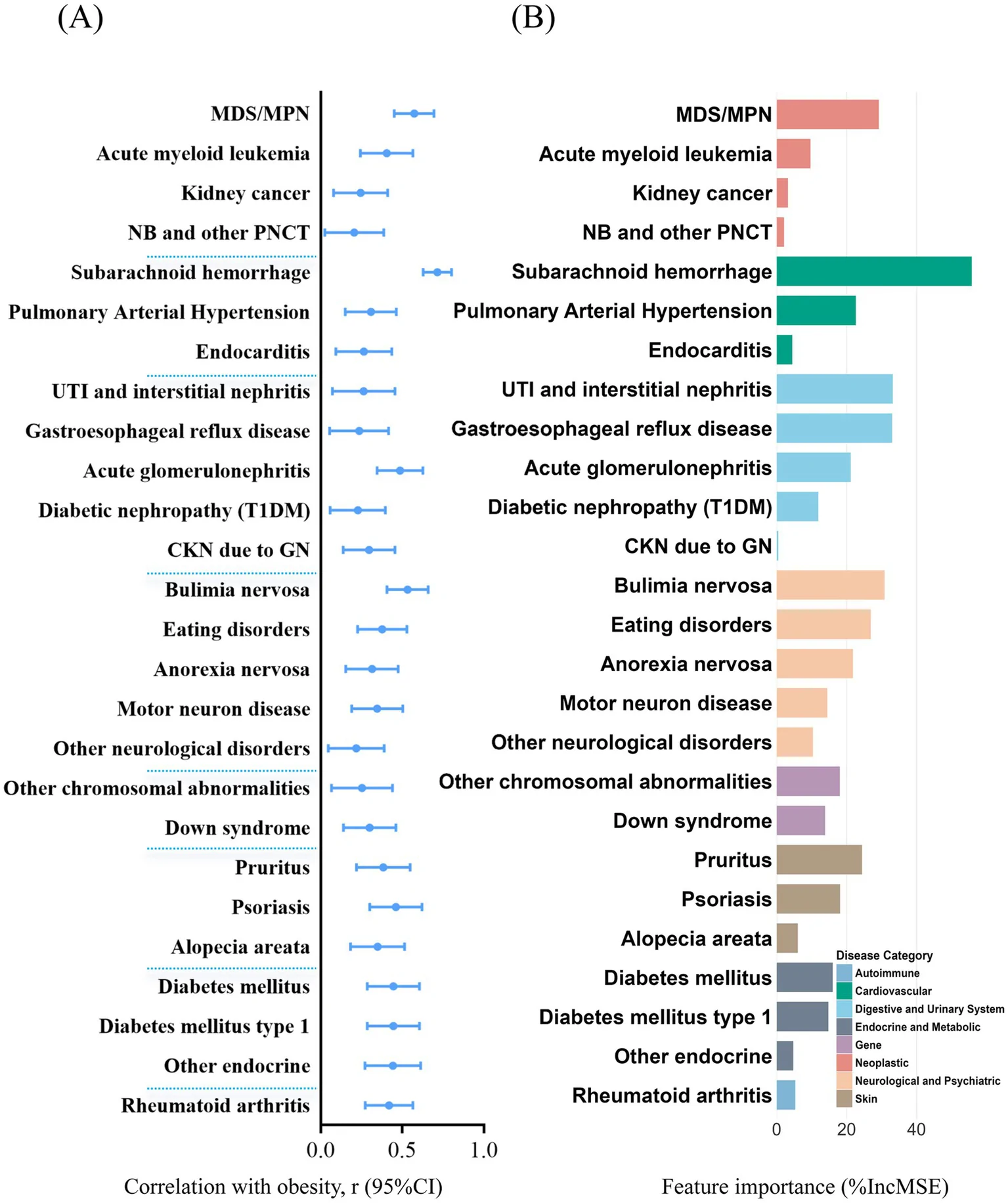
The association of obesity and NCDs, and random forest feature importance: Analyses stratified by gender. **(A)** Gender-stratified correlation analysis between obesity and NCDs. Only positive associations that remained statistically significant after FDR correction (*q* < 0.05) are shown. **(B)** Gender-stratified Random Forest feature importance for the obesity-NCDs associations. No positive associations remained statistically significant among girls after FDR correction. MDS/MPN: Myelodysplastic, myeloproliferative, and other hematopoietic neoplasms; CNS cancer: Brain and central nervous system cancer; UTI and interstitial nephritis: Urinary tract infections and interstitial nephritis; NB and other PNCT: Neuroblastoma and other peripheral nervous cell tumors; Cervical/uterine benign/in situ neoplasms: Benign and in situ cervical and uterine neoplasms; CKN due to GN: Chronic kidney disease due to glomerulonephritis; Diabetic nephropathy (T1DM): Chronic kidney disease due to diabetes mellitus type 1.

Stratified by sex, among males, 31 positive associations reached nominal significance, of which 26 remained statistically significant after FDR correction (Figure 4). The strongest correlations were observed for subarachnoid hemorrhage (*r* = 0.723, 95% CI: 0.624–0.799) and MDS/MPN (*r* = 0.581, 95% CI: 0.447–0.690; Figure 4A). Furthermore, subarachnoid hemorrhage (importance score: 57.81), UTI and interstitial nephritis (importance score: 33.21), gastroesophageal reflux disease (importance score: 32.99) were the highest importance scores (Figure 4B). Among females, three positive correlations reached nominal significance, but none remained statistically significant after FDR correction. Therefore, no Random Forest results are presented for girls.

Collectively, the sex-stratified analyses identified a larger number and broader range of country-level associations among boys than among girls.

## 4 Discussion

This study analyzed data from 174 countries worldwide, providing a detailed characterization of the global distribution of overweight and obesity among children aged 5–9 years, stratified by continent and by sex. Continental patterns revealed that the Americas, Europe, and the Western Pacific carried the heaviest overweight burden, while Africa and Southeast Asia had lower prevalence. Chile stood out as the nation with the highest childhood prevalence worldwide for both conditions: 63.47% for overweight and 35.75% for obesity. These regional and national extremes highlight the influence of socioeconomic, cultural, and dietary factors (24, 25). The high burden in the Americas and Western Pacific may be linked to Westernized diets and sedentary lifestyles (26–28), whereas the lower rates in Africa and Southeast Asia could be attributable to traditional food systems and higher energy expenditure (27, 29, 30). Chile’s top-ranking prevalence warrants urgent policy attention and further research into its unique combination of food environment, urbanization, and public health policies (31). The present study extends previous global analyses by examining country-level correlations between childhood overweight/obesity prevalence and a broad range of NCD prevalence outcomes.

Sex-stratified analyses revealed a male predominance in childhood obesity in the Americas and the Western Pacific, mirroring the continental patterns observed for overall overweight and obesity burdens. This male-predominant pattern raises questions regarding the drivers of sex-specific vulnerability (32, 33). These findings expand the understanding of weight-related health risks in children beyond traditional metabolic disorders, revealing the extensive systemic impact of overweight and obesity in early life (7, 34, 35). Notably, the correlation between overweight/obesity and NCDs is more pronounced in males, who also face a broader spectrum of correlated outcomes (36), highlighting gender-specific variations in the health risks posed by childhood weight abnormalities. Potential explanations include differential behavioral exposures in which boys show stronger and more extensive correlations between overweight/obesity and NCDs, including inflammatory conditions such as psoriasis. These differences are linked to sex-specific fat distribution and inflammatory responses in this age group (16, 17). Sex-stratified analyses identified a larger number of country-level associations among boys than among girls. This pattern should not be interpreted as evidence that individual boys are more susceptible to overweight or obesity-related diseases. Previous individual-level studies have reported sex-related differences in fat distribution, body composition, inflammatory responses, and behavioral exposures, which may provide possible hypotheses for the observed patterns (18–20). These findings highlight the necessity of gender-specific strategies for childhood weight management and NCD prevention.

Subsequently, we employed an ecological analysis to examine the associations between overweight/obesity and NCDs. Our results showed significantly positive associations between both overweight and obesity and a broad spectrum of NCDs among children aged 5–9 years, with different country-level patterns observed between boys and girls (1). At the global population level, childhood weight abnormalities are closely associated with multi-system disorders, with the strongest and most consistent associations observed for neoplastic diseases, digestive and urinary system disorders, neurological and psychiatric conditions, cardiovascular diseases, and skin diseases. Notably, subarachnoid hemorrhage showed the strongest correlation with both overweight and obesity. Given its rarity in children, this unexpected finding may reflect ecological bias, differences in surveillance, or residual confounding. Although inflammation, endothelial dysfunction, and altered hemodynamic regulation have been proposed as possible pathways, these mechanisms were not examined in the present study, and the finding should be regarded as hypothesis-generating (37, 38).

Moreover, among the identified associations, neoplastic diseases show the strongest correlation, especially hematological neoplasms such as myelodysplastic and myeloproliferative neoplasms, non-Hodgkin lymphoma, and acute myeloid leukemia. Recent pediatric evidence has primarily examined BMI in relation to treatment outcomes and prognosis among children already diagnosed with acute leukemia, rather than the incidence of hematological malignancies (39). Therefore, the present country-level correlations cannot establish that childhood overweight or obesity contributes to the development of these conditions. Correlations were also identified for digestive and urinary outcomes, including gastroesophageal reflux disease, urinary tract infections, and interstitial nephritis, as well as neurological, psychiatric, and cardiovascular outcomes. Previous studies have proposed chronic low-grade inflammation, metabolic dysregulation, endothelial dysfunction, and hemodynamic alterations as possible pathways linking excess adiposity with selected health outcomes (40). However, these mechanisms were not examined in the present ecological study, and some of the identified conditions lack well-established relationships with childhood overweight or obesity. These findings should therefore be interpreted cautiously as exploratory rather than causal.

However, this study also has inherent limitations associated with ecological research. First, as a cross-sectional analysis based on aggregate national-level data, it can only identify associations between childhood overweight/obesity and NCDs prevalence rather than establish causal relationships. Second, the ecological fallacy is a key constraint. Because the analysis was based on aggregated national-level data, the observed associations cannot be translated into individual-level disease risk (41). The findings should therefore be interpreted only as population-level ecological correlations. Third, the analyses were not adjusted for potentially important country-level confounders, including socioeconomic status, healthcare access, dietary patterns, physical activity, urbanization, and demographic characteristics. These factors may be associated with both childhood overweight/obesity prevalence and recorded NCD prevalence and may therefore partly explain the observed associations. In particular, differences in healthcare access and surveillance capacity may affect disease detection and reporting across countries. Therefore, the findings should be interpreted as unadjusted ecological correlations, and future studies should incorporate harmonized country-level covariates and individual-level longitudinal data. Fourth, the exclusion of countries with incomplete data may have introduced selection bias and limited the global representativeness of the findings.

## 5 Conclusion

Collectively, this study characterized the global distribution of overweight and obesity among children aged 5–9 years in 2022. Stratified by continent and sex, the highest prevalence occurred in the Americas, Europe, and Western Pacific, with Chile having the highest national rates (overweight: 63.47%; obesity: 35.75%). Males showed higher prevalence than females, especially in the Americas and Western Pacific. Weight abnormalities were significantly positively associated with various NCDs, most strongly with neoplastic, digestive/urinary, neurological/psychiatric, cardiovascular, and skin diseases; subarachnoid hemorrhage had the strongest correlation. Marked sex disparities were observed, with males showing more extensive associations. These findings describe country-level co-distribution patterns between childhood overweight/obesity and several NCD prevalence outcomes, with different patterns observed between boys and girls. They should be regarded as hypothesis-generating and may help guide global child health surveillance and future individual-level longitudinal research.

## Data Availability

Publicly available datasets were analyzed in this study. This data can be found at: https://vizhub.healthdata.org/gbd-results/, https://www.who.int/data/gho.
