# Supplementary material for: Global ecological associations between overweight/obesity and non-communicable disease prevalence among children aged 5–9 years: a sex-stratified analysis of 174 countries
Source: Front Public Health. 2026 Aug 26;14:1921938. doi: 10.3389/fpubh.2026.1921938 (PMC13553818; doi:10.3389/fpubh.2026.1921938)
Supplement: Supplementary file 1 [file Supplementary_file_1.docx]

**Figure S1. Flowchart of study design and analytical workflow.** National prevalence estimates of overweight and obesity among children aged 5-9 years were obtained from the WHO Global Health Observatory, whereas prevalence estimates of non-communicable diseases for the same age group were obtained from the Global Burden of Disease database. Countries were matched across the two databases, resulting in a final sample of 174 countries. NCDs outcomes with more than 90 valid country-level observations were retained for analysis. Six prespecified analytical groups were constructed according to weight status and sex, followed by Spearman correlation analysis, Benjamini-Hochberg false discovery rate correction, and exploratory disease-specific Random Forest regression.

**Figure S2.** Overview of prevalence distribution across global continents and countries. **A.** Country‑specific prevalence of overweight in children aged 5-9 years in 2022. **B.** Country‑specific prevalence of obesity in children aged 5-9 years in 2022. **C.** Continental prevalence of overweight and obesity in children aged 5-9 years.

**Figure S3.** Sex differences in obesity prevalence across continents.

**Figure S4.** Associations between overweight and NCDs in Children Aged 5-9 Years. **A.** Correlation analyses between overweight and NCDs. **B.** Random Forest feature importance of overweight across major disease categories. Only positive associations that remained statistically significant after FDR correction (*q* < 0.05) are shown. MDS/MPN: Myelodysplastic, myeloproliferative, and other hematopoietic neoplasms; CNS cancer: Brain and central nervous system cancer; Intestinal benign/in situ neoplasms: Benign and in situ intestinal neoplasms; UTI and interstitial nephritis: Urinary tract infections and interstitial nephritis; Neuroblastoma and other PNS tumors: Neuroblastoma and other peripheral nervous cell tumors.

**Figure S5.** Associations between obesity and NCDs in Children Aged 5-9 Years. **A.** Correlation analyses between obesity and NCDs. **B.** Random Forest feature importance of obesity across major disease categories. Only positive associations that remained statistically significant after FDR correction (*q* < 0.05) are shown. MDS/MPN: Myelodysplastic, myeloproliferative, and other hematopoietic neoplasms; CNS cancer: Brain and central nervous system cancer; Intestinal benign/in situ neoplasms: Benign and in situ intestinal neoplasms; UTI and interstitial nephritis: Urinary tract infections and interstitial nephritis; Neuroblastoma and other PNS tumors: Neuroblastoma and other peripheral nervous cell tumors; CKN due to GN: Chronic kidney disease due to glomerulonephritis; Diabetic nephropathy (T1DM): Chronic kidney disease due to diabetes mellitus type 1.
